# Lack of association of *CD44*-rs353630 and *CHI3L2*-rs684559 with pancreatic ductal adenocarcinoma survival

**DOI:** 10.1038/s41598-021-87130-0

**Published:** 2021-04-07

**Authors:** Manuel Gentiluomo, Chiara Corradi, Giuseppe Vanella, Astrid Z. Johansen, Oliver Strobel, Andrea Szentesi, Anna Caterina Milanetto, Péter Hegyi, Juozas Kupcinskas, Francesca Tavano, John P. Neoptolemos, Dania Bozzato, Thilo Hackert, Raffaele Pezzilli, Julia S. Johansen, Eithne Costello, Beatrice Mohelnikova-Duchonova, Casper H. J. van Eijck, Renata Talar-Wojnarowska, Carsten Palnæs Hansen, Erika Darvasi, Inna M. Chen, Giulia Martina Cavestro, Pavel Soucek, Liliana Piredda, Pavel Vodicka, Maria Gazouli, Paolo Giorgio Arcidiacono, Federico Canzian, Daniele Campa, Gabriele Capurso

**Affiliations:** 1 Department of Biology, University of Pisa, Via Derna 1, 56126 Pisa, Italy; 2 Pancreato-Biliary Endoscopy and Endosonography Division, Pancreas Translational and Clinical Research Center, IRCCS San Raffaele Scientific Institute, Milan, Italy; 3 Sant’Andrea Hospital, Faculty of Medicine and Psychology, Sapienza University of Rome, Rome, Italy; 4 Department of Oncology, Herlev and Gentofte Hospital, Copenhagen University Hospital, Herlev, Denmark; 5 Department of General, Visceral and Transplantation Surgery, University of Heidelberg, Heidelberg, Germany; 6 Institute for Translational Medicine, Medical School, University of Pécs, Pecs, Hungary; 7 First Department of Medicine, University of Szeged, Szeged, Hungary; 8 Department of Surgery, Gastroenterology, Oncology-Clinica Chirurgica, University of Padua, Padua, Italy; 9 Department of Gastroenterology and Institute for Digestive Research, Lithuanian University of Health Sciences, Kaunas, Lithuania; 10 Division of Gastroenterology and Research Laboratory, Fondazione IRCCS Casa Sollievo Della Sofferenza, San Giovanni Rotondo, Foggia, Italy; 11 Department of Medicine-Medicina Di Laboratorio, University of Padua, Padua, Italy; 12 Gastroenterology Unit, San Carlo Hospital, Potenza, Italy; 13 Department of Medicine, Herlev and Gentofte Hospital, Copenhagen University Hospital, Herlev, Denmark; 14 National Institute for Health Research Liverpool Pancreas Biomedical Research Unit, University of Liverpool, Liverpool, UK; 15 Department of Oncology, Faculty of Medicine and Dentristry, Palacky University Olomouc, Olomouc, Czech Republic; 16 Department of Surgery, Erasmus Medical Center, Erasmus University, Rotterdam, The Netherlands; 17 Department of Digestive Tract Diseases, Medical University of Lodz, Lodz, Poland; 18 Department of Surgery, Rigshospitalet, Copenhagen University Hospital, Copenhagen, Denmark; 19 Gastroenterology and Gastrointestinal Endoscopy Unit, Vita-Salute San Raffaele University San Raffaele Scientific Institute, Milan, Italy; 20 Laboratory of Pharmacogenomics, Biomedical Center, Faculty of Medicine in Pilsen, Charles University, Pilsen, Czech Republic; 21 ARC-Net Research Centre, University of Verona, Verona, Italy; 22 Department of the Molecular Biology of Cancer, Institute of Experimental Medicine, Czech Academy of Sciences, Prague, Czech Republic; 23 Laboratory of Biology, Medical School, National and Kapodistrian University of Athens, Athens, Greece; 24 Genomic Epidemiology Group, German Cancer Research Center (DKFZ), Heidelberg, Germany

**Keywords:** Cancer epidemiology, Cancer genetics, Cancer genomics, Gastrointestinal cancer, Oncology

## Abstract

Although pancreatic ductal adenocarcinoma (PDAC) survival is poor, there are differences in patients’ response to the treatments. Detection of predictive biomarkers explaining these differences is of the utmost importance. In a recent study two genetic markers (*CD44*-rs353630 and *CHI3L2*-rs684559) were reported to be associated with survival after PDAC resection. We attempted to replicate the associations in 1856 PDAC patients (685 resected with stage I/II) from the PANcreatic Disease ReseArch (PANDoRA) consortium. We also analysed the combined effect of the two genotypes in order to compare our results with what was previously reported. Additional stratified analyses considering TNM stage of the disease and whether the patients received surgery were also performed. We observed no statistically significant associations, except for the heterozygous carriers of *CD44*-rs353630, who were associated with worse OS (HR = 5.01; 95% CI 1.58–15.88; *p* = 0.006) among patients with stage I disease. This association is in the opposite direction of those reported previously, suggesting that data obtained in such small subgroups are hardly replicable and should be considered cautiously. The two polymorphisms combined did not show any statistically significant association. Our results suggest that the effect of *CD44*-rs353630 and *CHI3L2*-rs684559 cannot be generalized to all PDAC patients.

## Introduction

Pancreatic ductal adenocarcinoma (PDAC) is a lethal tumour type, with an increasing incidence over the past decades and a five year survival rate still as low as 9%^1^. PDAC is, therefore, projected to be the second cause of cancer-related deaths in the USA by 2030^2^ with a similar figure in most Western countries. One of the reasons of this meager survival is the lack of specific symptoms and of biological markers for early diagnosis and risk stratification. Moreover, treatment options are poor, and surgery in the setting of multimodal treatments is the only possible cure. However, there are marked differences in patients’ responses to the treatments, which cannot be explained by the traditional prognostic factors such as tumour size, lymph node involvement and distal metastasis^3^. Thus, investigation of biomarkers able to predict the tumour behaviour is of the utmost importance.

Genetic variability accounts for a large part of the risk to develop PDAC^4^. Several germline mutations, associated with well described hereditary syndromes, increase the risk of developing PDAC and subjects carrying such mutations are offered surveillance in the context of research protocols^5^. Besides these high penetrance germline mutations, there are overwhelming evidences on the role of germline genetic polymorphisms in the development of the disease, identified through genome-wide association studies (GWAS) or through large scale case–control studies^6–21^. However, the possible association between genetic variants and patients’ outcome in PDAC, both in terms of survival and response to treatments, has been poorly investigated with limited success^22–29^. The genetic contribution to PDAC survival has, however, been studied with GWAS. For example, a small-scale GWAS done on 252 PDAC cases, with subsequent validation done on 261 and 572 sets of patients, respectively, reported an association between a single nucleotide polymorphism (SNP) on chromosome 12 and overall survival (OS) of PDAC cases (*p* = 1.72 × 10^−7^ in the combined dataset)^27^. Innocenti et al. reported on a GWAS on OS of 351 pancreatic cancer patients treated with gemcitabine in the context of a randomized clinical trial. This study showed an association of a SNP in *IL17F* with OS (*p* = 9.51 × 10^−7^)^29^. Wu et al. performed the largest GWAS on OS to date in 1005 PDAC cases (10), of whom 642 were of European descent (from prospective cohort studies), used as discovery phase, and 363 retrospectively collected cases of Chinese descent used for replication^25^. In the first stage 131 SNPs at 28 loci showed association with OS of the PDAC cases (*p* < 10^−5^). Combining the discovery and the replication phases, a locus on chromosome 11 near the *SBF2* gene was the most significantly associated with OS of PDAC patients (*p* = 1.72 × 10^−7^). It is worth noticing that none of the findings of the above mentioned GWAS reached genome-wide significance level, conventionally set at *p* < 5 × 10^−8^, and that only a few loci were successfully replicated across multiple reports. In one of the few studies of this kind, we genotyped 44 SNPs proposed by the GWAS by Wu et al. in one of the largest study so far consisting in 1722 PDAC cases, in the context of the PANDoRA consortium^25^. We validated associations of one SNP in the *CTNNA2* gene (rs1567532) and one SNP in the *RUNX2* gene (rs12209785) with OS^26^. The lack of replicability of the PDAC survival loci is in striking contrast with the situation observed for GWAS-identified risk loci, which are generally confirmed in multiple independent studies, to some extent even in populations of diverse ethnic background.

Recently, Dimitrakopoulos and colleagues carried out a genome-wide screening of functional variants aimed at identifying novel markers associated with PDAC survival after pancreatic resection^30^. The authors used a dense SNP array and investigated more than 2 million SNPs in 331 PDAC patients in a two-phase approach. The main findings consisted in association of *CD44*-rs353630 and *CHI3L2*-rs684559 with PDAC survival. The diplotype combination of the two alleles of the two SNPs associated with better survival (C and G, respectively) reached genome-wide significance (HR = 0.38, 95%CI 0.27–0.53, *p* = 1.00 × 10^−8^) in the merged population consisting of the two phases.

One of the reasons behind the lack of success of studies investigating genetic variants as biomarkers of disease outcome is that most studies so far were underpowered and therefore prone to statistical fluctuation, and/or that a proper replication phase in an independent adequately sized population was lacking. Considering the capricious nature of association studies, we aimed at validating the findings by Dimitrakopoulos and colleagues in a large cohort of 1856 PDAC cases among which 685 resected patients with tumour stage I or II from the PANcreatic Disease ReseArch (PANDoRA) consortium.

## Results

### Population description and data filtering

The population investigated in the present study consisted overall of 1856 PDAC patients: 105 with stage I, 717 with stage II, 348 with stage III and 686 with stage IV at the time of diagnosis. The median age was 66 and both sexes were represented with a slight majority of males (56%). 685 patients with stage I or II were operated with curative intent. A description of the population is shown in Table 1.

**Table 1:** Description of the study population.

Country	Patients	Sex (%)		Stage				Age (years)
		Females	Males	I	II	III	IV	Median (Q_1_–Q_3_)
Italy	483	211	272	18	188	86	191	67 (60–74)
Denmark	431	189	242	17	149	64	201	68 (62–73)
Germany	351	136	215	14	244	28	65	65 (58–70)
Hungary	254	130	124	18	16	120	100	65 (59–73)
Czech Republic	151	63	88	27	69	19	36	62 (58–69)
Lithuania	87	41	46	1	21	13	52	69 (60–73)
Poland	46	26	20	8	15	6	17	61 (54–66)
Romania	30	11	19	2	0	10	18	63 (54–67)
The Netherlands	15	6	9	0	10	2	3	68 (60–76)
United Kingdom	8	5	3	0	5	0	3	69 (65–73)
Total	1856	818	1038	105	717	348	686	69 (59–72)

The genotyping call rate was 95% for *CD44*-rs353630 and 96% for *CHI3L2*-rs684559, the concordance between the duplicated samples was higher than 99%. All the SNPs resulted to be in HWE equilibrium with non-significant *p* values (*p* > 0.05) when considering all the individuals together or dividing them by country of origin. The minor frequency (MAF) observed in PANDoRA is 25.62% for *CD44*-rs353630 and 36.07% for *CHI3L2*-rs684559, in line with what observed in the European populations in the 1000 Genomes project: 25.35% for *CD44*-rs353630 and 36.88% for *CHI3L2*-rs684559.

### Survival analysis

**Figure 1:**
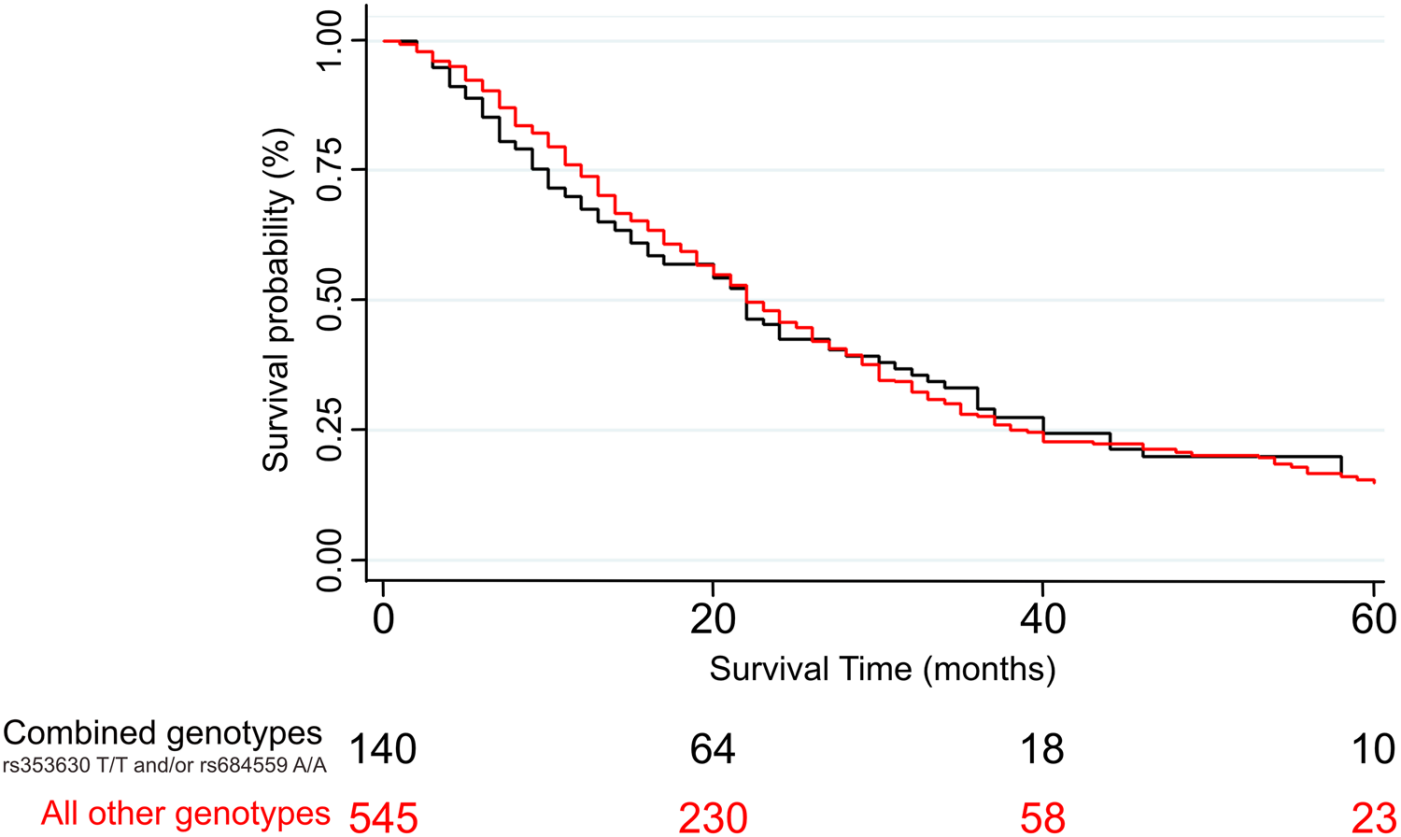
Kaplan–Meier curve showing the survival of resected PDAC patients diagnosed in stage I or II, according to the combined genotypes of CD44-rs353630 and CHI3L2-rs684559.

Analysing the whole PDAC case population and adjusting for age, sex and stage we observed no statistically significant association between the two SNPs under investigation and OS of PDAC patients, with the best finding consisting in a non-significant trend for the C/C genotype of the *CD44*-rs353630 of having a worse survival compared with the T/T homozygous (HR = 1.28, 95% CI 0.98–1.68, *p* = 0.074). We also performed stratified analysis by disease stage and when analysing the individuals that presented stage I disease at diagnosis we observed a statistically significant association for the heterozygous C/T carriers of *CD44*-rs353630 and worse OS compared with the reference (HR = 5.01; 95% CI 1.58–15.88; *p* = 0.006). The analysis considering only the 685 patients who received surgery did not show any statistically significant associations. We also performed an analysis considering the combination of the two markers and we observed no statistically significant result. The frequencies and distribution of the genotypes, the HR and 95% CI for the association with PDAC survival are shown in Table 2. A Kaplan–Meier curve with the survival of resected PDAC patients diagnosed in stage I or II (*p* = 0.64), according to the combined genotypes of the two SNPs is showed in Fig. 1. We performed additional analyses stratifying by country of origin. We did not observe any statistically significant associations, with the exception of a longer OS for patients from Hungary diagnosed with stage I or II PDAC and heterozygous for the *CHI3L2*-rs684559 SNP, in comparison with the homozygotes for the common allele (HR = 0.19; 95% CI 0.05–0.71; *p* = 0.014).

**Table 2:** Survival analysis of *CD44*-rs353630 and *CHI3L2*-rs684559.

SNP	All patients^a^			All patients^b^		Stage I^c^		
	No.	HR (95% CI)	*p* value	HR (95% CI)	*p* value	No.	HR (95% CI)	*p* value
**rs353630**								
T/T	134	1 [Reference]	NA	1 [Reference]	NA	13	1 [Reference]	NA
C/T	683	1.16 (0.94–1.44)	0.165	1.15 (0.93–1.43)	0.206	34	5.01 (1.58–15.88)	**0.006**
C/C	1039	1.13 (0.92–1.39)	0.259	1.13 (0.91–1.39)	0.258	58	2.20 (0.73–6.61)	0.162
C/T + C/C	1722	1.14 (0.93–1.40)	0.202	1.14 (0.93–1.40)	0.220	92	2.77 (0.95–8.06)	0.062
Resected								
T/T	85	1 [Reference]	NA	1 [Reference]	NA	9	1 [Reference]	NA
C/T	372	1.21 (0.92–1.61)	0.178	1.23 (0.93–1.63)	0.152	25	4.43 (0.91–21.64)	0.066
C/C	630	1.26 (0.96–1.65)	0.098	1.28 (0.98–1.68)	0.074	51	2.34 (0.51–10.62)	0.272
C/T + C/C	1002	1.24 (0.95–1.62)	0.111	1.26 (0.97–1.65)	0.087	76	2.70 (0.61–11.91)	0.190
**rs684559**								
A/A	256	1 [Reference]	NA	1 [Reference]	NA	11	1 [Reference]	NA
G/A	827	1.01 (0.86–1.19)	0.918	0.97 (0.82–1.14)	0.719	44	0.50 (0.21–1.20)	0.119
G/G	773	0.96 (0.82–1.13)	0.640	0.94 (0.80–1.11)	0.456	50	0.54 (0.23–1.26)	0.155
G/A + G/G	1600	0.99 (0.85–1.15)	0.849	0.96 (0.82–1.11)	0.558	94	0.52 (0.24–1.16)	0.110
Resected								
A/A	157	1 [Reference]	NA	1 [Reference]	NA	6	1 [Reference]	NA
G/A	479	0.99 (0.81–1.24)	0.984	0.97 (0.78–1.20)	0.777	38	0.61 (0.19–1.94)	0.407
G/G	451	1.00 (0.81–1.24)	0.981	0.98 (0.79–1.22)	0.860	41	0.59 (0.19–1.89)	0.376
G/A + G/G	930	1.00 (0.82–1.22)	0.999	0.97 (0.80–1.19)	0.804	79	0.60 (0.20–1.81)	0.367
**Combined SNPs**^**d**^								
rs353630 T/T and/or rs684559 A/A	368	1 [Reference]	NA	1 [Reference]	NA	20	1 [Reference]	NA
All other genotypes	1488	1.04 (0.91–1.18)	0.582	1.02 (0.89–1.16)	0.826	85	1.38 (0.65–2.91)	0.403
Resected								
rs353630 T/T and/or rs684559 A/A	229	1 [Reference]	NA	1 [Reference]	NA	12	1 [Reference]	NA
All other genotypes	858	1.06 (0.90–1.27)	0.480	1.06 (0.89–1.26)	0.526	73	1.5 (0.55–4.12)	0.431

SNP	Stage II^c^			Stage I and II^c^				
	No.	HR (95% CI)	*p* value	No.	HR (95% CI)	*p* value		
**rs353630**								
T/T	47	1 [Reference]	NA	60	1 [Reference]	NA		
C/T	265	1.08 (0.73–1.59)	0.709	299	1.27 (0.88–1.83)	0.203		
C/C	405	1.07 (0.73–1.56)	0.730	463	1.17 (0.82–1.67)	0.388		
C/T + C/C	670	1.07 (0.74–1.55)	0.714	762	1.20 (0.85–1.71)	0.295		
Resected								
T/T	44	1 [Reference]	NA	53	1 [Reference]	NA		
C/T	209	1.08 (0.72–1.62)	0.721	234	1.23 (0.83–1.81)	0.311		
C/C	347	1.15 (0.78–1.71)	0.476	398	1.23 (0.84–1.79)	0.291		
C/T + C/C	556	1.12 (0.77–1.65)	0.550	685	1.23 (0.85–1.77)	0.282		
**rs684559**								
A/A	102	1 [Reference]	NA	113				
G/A	333	0.93 (0.71–1.22)	0.617	377	0.87 (0.67–1.12)	0.270		
G/G	282	0.92 (0.70–1.22)	0.569	332	0.89 (0.68–1.15)	0.364		
G/A + G/G	615	0.93 (0.72–1.20)	0.567	709	0.88 (0.69–1.11)	0.278		
Resected								
A/A	89	1 [Reference]	NA	95	1 [Reference]	NA		
G/A	276	0.89 (0.67–1.19)	0.438	314	0.88 (0.66–1.16)	0.358		
G/G	235	0.95 (0.71–1.28)	0.739	276	0.93 (0.70–1.23)	0.606		
G/A + G/G	511	0.92 (0.70–1.20)	0.539	590	0.90 (0.69–1.17)	0.432		
**Combined SNPs**^**d**^								
rs353630 T/T and/or rs684559 A/A	144	1 [Reference]	NA	164	1 [Reference]	NA		
All other genotypes	573	0.92 (0.74–1.16)	0.489	658	0.95 (0.77–1.17)	0.623		
Resected								
rs353630 T/T and/or rs684559 A/A	128	1 [Reference]	NA	140	1 [Reference]	NA		
All other genotypes	472	0.93 (0.73–1.19)	0.571	545	0.97 (0.77–1.22)	0.780		

NA, not applicable; HR, Hazard Ratio, CI, confidence interval. Statistically significant results (*p* < 0.05) are in bold. Numbers may not add up to the total number of subjects in the respective stratum due to missing data.

^a^The analyses were adjusted by tumour stage (stages I through IV).

^b^The analyses were adjusted by tumour stage, country of origin, sex and age.

^c^The analyses were adjusted by, country of origin, sex and age.

^d^The combined analysys was performed using the rs353630 T/T and rs684559 A/A genotypes as reference.

### In silico functional characterization

The GTEx portal identified only one eQTL for *CD44*-rs353630: the weak (*p* = 0.00025) association between the A allele and a decreased expression of the *CD44* gene in the tibial nerve. GTEx predicts that *CHI3L2*-rs684559 is an eQTLs for the *CHI3L2, DENND2D, CHIAP2* and *CHIA* genes across seven tissues, although none of them in the pancreas (p-values ranging from 0.000026 to 3.7 × 10^−20^). For *CD44*-rs353630 RegulomeDB showed a rank of 4 that is the third lowest and indicates that the polymorphism may be situated in a possible transcription factor binding site. On the other hand, for *CHI3L2*-rs684559 RegulomeDB showed a rank of 1b indicating a very high degree of supporting data in the database and high probability of functional relevance. Haploreg showed no SNPs in LD with *CD44*-rs353630 and no eQTLs, while six SNPs (rs2764546, rs12048900, rs12024633, rs2494004, rs1325284, rs2494006) in LD with *CHI3L2*-rs68455 and 14 eQTLs in lymphoblastoid cell lines and in the whole blood.

## Discussion

In the last decades genetic epidemiological approaches, especially those performed through genome-wide association studies in the context of multicentric studies, have identified thousands of SNPs and have shed light on the biology of many cancer types. As far as regards PDAC, around 30 risk loci showed a clear association with the development of the disease. However, differently from other cancer types such as breast, lung, prostate, colorectal cancers, and multiple myeloma^31–37^, the involvement of genetic variability in PDAC survival has been investigated with limited success. The most reliable factors in determining PDAC patient survival are, therefore, clinical parameters such as TNM stage, tumour grade, margins of resection and pre- and postoperative levels of C-reactive protein (CRP) and CA-19-9^38^. Unfortunately, these factors have limited accuracy in predicting the outcome of PDAC patients and their response to treatments. Therefore, the identification of additional biological markers would be of the utmost importance as a basis to elaborate novel decisional algorithms aimed at optimizing treatment and improving patients' outcomes. Additionally, discovering new PDAC survival genetic loci may give insight to the molecular mechanisms of survival and suggest, directly or indirectly, new therapeutic targets.

Several SNPs have been proposed to be possible prognostic markers in PDAC patients, given the association with survival. However, with only a few exceptions, none has been consistently replicated in following studies^25–29^. Recently two novel candidates, identified through a GWAS approach, have been proposed. A total of 331 PDAC patients were investigated by means of a dense SNP array, encompassing more than 2 million SNPs, finding an association between *CD44*-rs353630 and *CHI3L2*-rs684559 and PDAC survival in resected patients of whom 90.9% with stage I or II. The diplotype combination of these two alleles was, indeed, associated with longer survival after surgery^30^. We genotyped these two SNPs and analysed them, separately or in combination, in a large set of PDAC cases belonging to the PANDoRA consortium. Considering the paucity of evidences on markers related to PDAC survival this attempt to generalize the results of the two SNPs was necessary. We had more than 99% power to replicate the associations, but we observed none when analysing the whole population or when considering only the resected patients. The most clinically relevant result of the study by Dimitrakopoulos and colleagues is the association of the two combined polymorphisms with better survival in 331 patients resected with intent of radicality. Availability of such a biomarker might be extremely relevant as it would help selection of patients to consider for upfront surgery or neoadjuvant chemotherapy, thus contributing to a personalized medicine approach for PDAC. However, in our subgroup analysis on 685 patients resected with stage I or II disease (therefore with a much larger sample size and a power to detect the association greater than 99%) the same SNP combination did not show any statistically significant association (the best association we observed had a *p* = 0.48).

Further stratifying the population by stage, we observed a statistically significant association between *CD44*-rs353630 C/T individuals and worse OS among PDAC cases diagnosed with stage I. The estimate is very high (HR > 5) and it goes in the opposite direction of the ones reported by Dimitrakopoulos, which all showed association of the effect alleles/genotypes with better OS. These findings arise from an analysis conducted in a very small subgroup of individuals and only comparing heterozygous with the reference homozygous (N = 34 vs. 13, respectively) and therefore must be taken with caution. The analysis conducted to assess the functional relevance of the SNPs clearly shows that for *CD44*-rs353630 there is little or no evidence of its involvement in gene expression, while *CHI3L2*-rs684559 is possibly an eQTL with regulatory potential in several tissues, but not in the pancreas. It is therefore difficult to link the SNPs to a functional role in pancreatic cancer progression or outcome.

Analyses stratified by country of origin did not show anything remarkable, with the exception of a weak association with longer OS in Hungarian patients diagnosed in stage I or II for heterozygous for the *CHI3L2*-rs684559 SNP. We note that this association is not significant if we consider a significance threshold adjusted for multiple testing (the observed association had *p* = 0.014, but analyses stratified by 10 countries of origin have an adjusted threshold of significance of *p* = 0.05/10 = 0.005). Considering also the relatively small number of cases used for this analysis (18 patients diagnosed with stage I and 16 in stage II), and the difficulty of explaining why the polymorphism should be associated with OS only in Hungarian patients, we are inclined to think that this result is due to statistical fluctuation and does not reflect a real association. A possible limitation of the present study is the lack of information on chemotherapeutic regimens administered to the patients, either before or after surgery. This prevents us to explore association between the genetic variability and drug response that may help personalization of treatments. In addition, the included patients were not consecutively enrolled in each center, and that could add a potential bias to the findings. We believe, however that given the samples size this bias should be diluted and unlikely to be responsible for the lack of statistically significant association that we observed.

## Material and methods

### Study population

The PANDoRA consortium consists of a multicentric study that includes 12 European countries (Italy, Germany, Poland, United Kingdom, Hungary, Czech Republic, Greece, Lithuania, the Netherlands, Denmark, Ukraine, Romania) and Japan and has been described in detail elsewhere^9^.

Briefly, all cases included in the consortium population are defined by a confirmed diagnosis of pancreatic cancer and were collected with a non-consecutive sampling approach in 21 different centers, including departments of surgery, endoscopy and gastroenterology. In addition to pancreatic cancer cases, controls have been selected among the general population, blood donors and among hospitalized subjects with different diagnosis excluding cancer. In the present study, 1856 PDAC cases of European descent were included, for whom information on country of origin, sex, age at diagnosis, overall survival (OS) and disease stage assessed by TNM stage was available. Disease stage was defined according to American Joint Committee on Cancer TNM system 7th version^39^, and patients were categorized as stage I (T1-2, N0, M0), stage II (T3, N0, M0/T1-3, N1, M0), stage III (T4, any N, M0), stage IV (any T, any N, M1). The patients that underwent surgery were categorized by the stage at the time of operation.

All subjects gave their informed consent for inclusion before they participated in the study. The study was conducted in accordance with the Declaration of Helsinki, and the protocol was approved by the Ethics Commission of the Medical Faculty of the University of Heidelberg (project identification code: S-565/2015). A description of the population is shown in Table 1.

### SNPs selection, sample preparation and genotyping

We selected two germline variants, *CD44*-rs353630 and *CHI3L2*-rs684559 that were proposed recently by Dimitrakopoulos and colleagues as associated with survival of PDAC patients that received surgery. For each subject DNA was extracted from whole blood using a QIAamp 96 DNA QIAcube HT Kit on QIAcube 96 instrument and kept frozen at − 20° till use. Genotyping was conducted using TaqMan (ABI, Applied Biosystems, Foster City, CA, USA) allelic discrimination technology following the manufacturer’s recommendation (assay id C____779802_10 for *CD44*-rs353630 and assay id C___3138574_20 for *CHI3L2*-rs684559). For each reaction 10 ng of dried DNA were used. The genotyping was conducted in 384-well plates and duplicated samples (8%) were included in each plate for quality control purpose. The identity of the samples was not known to the personnel performing the lab work. Genotype attribution was made using QuantStudioTM 5 Real-Time PCR system (Thermofisher, USA) and QuantStudio software.

### Statistical analysis

Deviation from Hardy–Weinberg equilibrium (HWE) was tested for both polymorphisms in the overall population and dividing for country of origin using the Pearson corrected chi square test. Survival analysis was conducted by Cox proportional hazard models with OS as endpoint, computing hazard ratios (HR) and 95% confidence intervals (CI), using an additive and a codominant inheritance models and setting the same alleles used by Dimitrakopoulos and colleagues as the reference category (T for *CD44*-rs353630 and A for *CHI3L2*-rs684559). In addition, considering that the most statistically significant association observed by Dimitrakopoulos was the one combining the two genotypes, we also performed this analysis defining two groups: the individuals with the *CD44*-rs353630 T/T and/or *CHI3L*2-rs684559 A/A as the reference category and all the other genotype combinations as the other group. For this analysis we considered only individuals with 100% call rate. All analyses were adjusted for sex, age and TNM stage. We also performed an analysis adjusting only for disease stage, since Dimitrakopoulos and colleagues did not adjust for sex and age.

As the vast majority (90.9%) of the patients in the study by Dimitrakopoulos and colleagues were resected with stage I or II, stratified analysis was conducted considering patients showing TNM stage I, TNM stage II, and TNM stage I and II together. We also performed a stratified analysis considering TNM stage I and II for patients who received surgery, as well as by country of origin. The probability of survival was calculated by means of a Kaplan–Meier curve.

### Functional analysis using bioinformatic tools

To test the possible functional relevance of *CD44*-rs353630 and *CHI3L2*-rs684559 we used several bioinformatic tools. In particular, the Genotype-Tissue Expression (GTEx) project portal (https://gtexportal.org/home/) was used to identify potential cis-acting expression quantitative trait loci (eQTLs) in order to establish whether the two SNPs could be involved in the expression of nearby genes (accession date 8th May 2020)^40^. We analysed all the tissues present in the database. We have used Regulome DB 2.0 (https://www.regulomedb.org/regulome-search/) to evaluate the effect of the SNPs with regulatory regions of the human non-coding genome^41^. Regulome DB 2.0 assigns to each SNP a rank consisting of 14 steps, that reflect the volume of the supporting data on the functional relevance of the SNP, going from 6 (lowest amount of evidence) to 1a (highest amount of evidence). Finally, we used Haploreg v4.1 (https://pubs.broadinstitute.org/mammals/haploreg/haploreg.php) to link the SNPs to functional annotations of the genome^42^. Haploreg is designed to explore the possible mechanistic interaction of the candidate SNPs (and all SNPs in LD with them) with regulatory motifs.

### Ethics declarations

The study was conducted in accordance with the Declaration of Helsinki, and the protocol was approved by the Ethics Commission of the Medical Faculty of the University of Heidelberg (project identification code: S-565/2015).

## Conclusions

In conclusion, we attempted to replicate the results of a genome-wide investigation on genetic polymorphisms and survival of PDAC patients, with a well-powered study. Our findings suggest no involvement in the PANDoRA population of the two variants reported by Dimitrakopoulos and colleagues and highlight a lack of functional or regulatory role of *CD44*-rs353630 and *CHI3L2*-rs684559 in the pancreatic tissue. Our results strongly support the need to consider with caution findings on genetic variables as prognostic markers in PDAC, in the absence of replication in large and independent datasets or of compelling in vitro mechanisms to support the associations.

Moreover, it would be desirable for future studies to have information on the therapy and to include this information in the analyses. This could make possible to evaluate the different genetic predisposition in drug response, and further our knowledge of this deadly disease.

## Data Availability

Owing to ethical and legal reasons, raw data of this work are not publicly deposited. The PANDoRA primary data for this work will be made available to researchers who submit a reasonable request to the corresponding author, conditional to approval by the PANDoRA Steering Committee and Ethics Commission of the Medical Faculty of the University of Heidelberg. Data will be stripped from all information allowing identification of study participants.
